# Intraosseous access in neonates is feasible and safe – An analysis of a prospective nationwide surveillance study in Germany

**DOI:** 10.3389/fped.2022.952632

**Published:** 2022-07-26

**Authors:** Eva Schwindt, Daniel Pfeiffer, Delphina Gomes, Sebastian Brenner, Jens-Christian Schwindt, Florian Hoffmann, Martin Olivieri

**Affiliations:** ^1^Department of Pediatrics and Adolescent Medicine, Division of Neonatology, Pediatric Intensive Care and Neuropediatrics, Comprehensive Center for Pediatrics, Medical University Vienna, Vienna, Austria; ^2^Pediatric Intensive Care Unit, Dr. von Hauner Children’s Hospital, Ludwig-Maximilians-University Munich, Munich, Germany; ^3^Department of Pediatrics, Division of Neonatology and Pediatrics Intensive Care, University Hospital Carl Gustav Carus, Dresden, Germany; ^4^Pediatric Working Group, Austrian Resuscitation Council, Graz, Austria

**Keywords:** intraosseous access, neonatal, resuscitation, emergency access, delivery room management, vascular access

## Abstract

**Background:**

This was a prospective surveillance study to investigate reports on the safety and frequency of use of intraosseous (IO) access in neonates.

**Methods:**

Over a two-year period, paediatric hospitals in Germany were asked to report all cases of IO access to the nationwide Surveillance Unit for Rare Paediatric Diseases (ESPED). Hospitals reporting a case submitted responses *via* an anonymised electronic questionnaire, providing details on indication, success rate, system used, location, duration to first successful IO access, complications, alternative access attempts and short-term outcome. We present a subset of data for IO use in infants of less than 28 days.

**Results:**

A total of 161 neonates (145 term and 16 preterm born infants) with 206 IO access attempts were reported. In 146 neonates (91%), IO access was successfully established, and success was achieved with the first attempt in 109 neonates (75%). There was no significant impact of gestational age or provider’s educational level on success rates. In 71 infants with successful IO access (79%), the estimated duration of placement was less than 3 min. The proximal tibia was the predominant site used. A semiautomatic battery-driven device was used in 162 attempts (88%). The most often applied medications via IO access were crystalloid fluid and adrenaline. Potentially severe complications occurred in 9 patients (6%).

**Conclusion:**

Within this surveillance study, IO access in neonates was feasible and safe. IO access is an important alternative for vascular access in neonates.

## Introduction

Establishing vascular access in neonates during cardiopulmonary resuscitation is a challenge, even for experienced paediatricians and neonatologists. Resuscitation events requiring epinephrine administration immediately after birth are rare, accounting for approximately 0.05% of births (1). However, approximately one in four emergency department visits in the neonatal period (< 28 days of life) are due to severe conditions that require immediate venous access (2). Intraosseous access (IO) offers a fast and reliable method for achieving emergency access for fluids and drugs when peripheral venous access fails (1, 3–5). Severe complications seem to be rare but include tibial fracture (6), haematoma (7), osteomyelitis (8), and extravasation of fluids and medications resulting in necrosis with compartment syndrome (9) as well as subsequent amputation (10). For adults and children beyond the neonatal setting, IO access is recommended as a first-line method of establishing vascular access in cardiac arrest and peri-arrest settings (11).

Regarding the population of neonates, currently, an increasing number of publications provide evidence that intraosseous placement is possible and can be effective in full-term and even preterm newborns (5, 7, 12). A recent animal study showed equal distributions and effects of intraosseous administration of epinephrine compared to intravenous administration in newborn lambs (13). In the recently published 2021 guidelines on newborn life support from the European Resuscitation Council, IO access is mentioned for the first time as an alternative possibility for emergency venous access alongside the umbilical venous catheter (UVC) (1). Nevertheless, to date, data on the feasibility and short-term safety of IO placement in neonates are lacking. The aim of this study was to present real-life data on the safety and feasibility of IO access in neonates up to 28 days of life. We present subgroup data of neonates taken from a 2-year prospective surveillance study on paediatric resuscitation events in Germany (Surveillance Unit for Rare Paediatric Diseases, ESPED).

## Materials and methods

### Data collection

ESPED is a well-established hospital-based German-wide prospective surveillance system for rare paediatric diseases, and its method of operation was previously published (14–16). Regular analyses of its capture rates showed that the completion of ESPED surveillance system reports is consistently greater than 90% (14). A two-year prospective surveillance study on the use of IO access in paediatric emergency medicine was performed from July 2017 to June 2019 including 345 children’s hospitals or paediatric departments, which constitutes the basis of this study. All data available in the ESPED database concerning IO access attempts in neonates during the first 28 days of life were included in this analysis.

German paediatric hospitals and departments involved in the ESPED surveillance study received a monthly reminder (via mail) to report any case of IO access, including those performed in their centre as well as those arriving with an IO needle from another centre or from out of hospital. The report of a case prompted a study-specific pseudonymized questionnaire that was designed by a consensus panel of paediatric emergency specialists. The questionnaire included demographic data, patient diagnosis and outcome, indication and details on IO access use. Success for IO access was defined as functioning IO access, which was assessed by the infusion of fluids/drugs. The duration of IO establishment was estimated retrospectively and defined as the time from the decision to the first infusion.

The questionnaire was completed by the responsible physician or, if this was not possible, by someone who had attended the event. If IO access was established out of the hospital, the responsible physician at the hospital the patient was transferred to completed the questionnaire. All reports were checked for plausibility of data by the principal investigator.

### Statistical analysis

Data were entered into a Microsoft Office Access 2003 (Washington, United States; Version 11.0) database and transferred to R statistical package (R Foundation for Statistical Computing, Vienna, Austria, version 3.6.2) for group comparisons. We compared patient age (0–24 h, 1–28 days) by doctor’s education level (chief/senior physician or resident/assistant physician), systems used, the number of IO-access attempts, success of IO establishment, complications and the number of alternative venous access attempts using Student’s *t*-test and χ^2^ test as appropriate. Missing data are indicated for each subject. All numbers are rounded up.

## Results

The overall response rate (after a case with IO access was reported and a questionnaire was sent) was 94%. A total of 161 neonates (145 term and 16 preterm born infants) with 206 IO-access attempts were reported to ESPED during the study period, and information was provided via questionnaire. Seventy-eight were male (49%; 3 unknown). The mean gestational age (GA) was 39 + 0 (range 25 + 6 to 42 + 0). Overall, 103 neonates (64%) survived to hospital discharge.

In 113 neonates (70%), IO access was established during the first 24 h of life. In 48 neonates (30%), IO access was established in a period up to 28 days. Diagnoses for both age groups are listed in Table 1. Table 2 summarises the details of the analysed cases with neonatal IO access regarding puncture site, IO device used, estimated time for IO establishment and previous alternative access attempts.

**TABLE 1:** Diagnoses preceding IO access in neonates.

Diagnosis (indication for IO)	Total (%)	< 24 h	24 h to 28 days
Arrhythmia	5 (3.1)	0	5
of which required CPR	2 (40.0)	0	2
Congenital heart defects	17 (10.6)	7	10
of which required CPR	5 (29.4)	4	1
Perinatal asphyxia	98 (60.9)	98	0
of which required CPR	66 (67.4)	66^1^	0
Respiratory insufficiency	10 (6.2)	5	5
of which required CPR	1 (10.0)	0^1^	1
Shock or Sepsis	9 (5.6)	2	7
of which required CPR	2 (22.2)	2	0
Resuscitation (CPR)^2^	16 (9.9)	0	16
Other^3^	6 (3.7)	1	5
Total	161 (100)	113	48

Values presented are n.

^1^One case unknown.

^2^Underlying pathology unknown.

^3^One case each of hyperammonaemia, intoxication, perinatal acidosis, poor peripheral vein status, seizure, and urgent surgery/anaesthesia.

**TABLE 2:** Detailed information on IO accesses analysed in 161 neonates, 146 successful IO accesses and 206 IO attempts.

Variable	Count (n)	Proportion (%)^1^
**IO accesses (information available for 161 of 161 neonates)**		
Successful	146	91
Unsuccessful	15	9
**Location of IO establishment (information available for 161 of 161 neonates)**		
Reporting hospital	102	63
Out of reporting hospital	59	37
**Site of IO access (information available for 202 of 206 attempts)**		
Prox. tibia	192	95
Dist. tibia	3	2
Dist. femur	4	2
Prox. humerus	2	1
Left side (information available for 200 attempts)	103	52
Right side (information available for 200 attempts)	97	49
**System used (information available for 185 of 206 attempts)**		
EZIO (Teleflex, United States)	162	88
EZIO inserted manually	4	2
COOK (Cook Medical, United States)	17	9
B.I.G (Persys Medical, United States)	2	1
**Estimated time for IO establishment (information available for 90 of 146 neonates with successful IO access)**		
> 3 min	19	21
< 3 min	71	79
2–3 minutes	17	24
1–2 min	36	51
< 1 min	18	25
**Medication administered (information available for 146 of 146 neonates with successful IO access)**		
Crystalloids	139	91
Adrenaline	81	53
Sedation	19	13
Others^2^	95	65
**Functioning of IO access (information available for 146 of 146 neonates with successful IO access)**		
Worked until intended removal	126	86
Accidental dislocation	20	14
**Removal of IO access (information available for 136 of 146 successful IO attempts)**		
< 6 h	86	63
< 24 h	110	81
> 24 h	5	4
After unsuccessful resuscitation	21	15
**Alternative access attempts before IO decision-making (information available for 137 of 161 neonates)**		
No alternative access attempt	28	20
One or more alternative access attempts	109	80
Peripheral venous catheter attempts	96	88
Umbilical venous catheter attempts	22	20
Central venous catheter attempts	4	4
**Complications (information available for 155 of 161 neonates with IO attempts)**		
No complication	100	65
Complication observed	55	35
Minor complication	46	84
Potentially severe complication	9	16

All numbers are rounded up.

^1^Percentage in relation to the corresponding number of cases (each stated separately).

^2^The following medications were administered (number of patients who received this medication indicated in brackets): sodium bicarbonate (12), blood products (12), antibiotics (12), analgesia (12), glucose (9), vasoactive therapy (9), alprostadil (9), induction of anaesthesia (6), antiepileptic drugs (2), amiodarone (2), akrinor (cafedrine/theodrenaline; 1), atropine (1), calcium (1), colloidal fluids (2), glucagon (1), hydrocortisone (1), magnesium (1), tris-buffer (1) and vitamin k (1).

### Success rates

**FIGURE 1:**
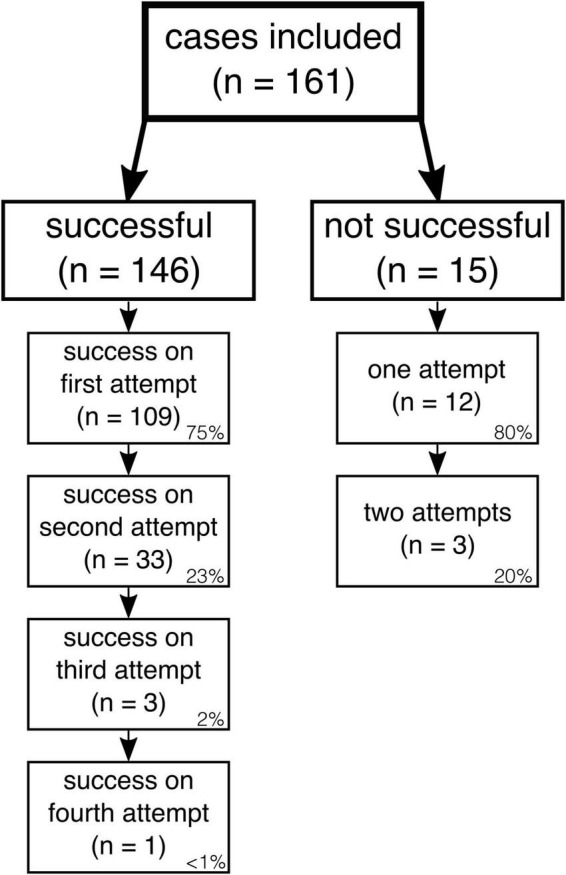
IO access attempts and success rates in the total study population (*n* = 161). Relative proportions of successful and unsuccessful attempts are given on the bottom right of each box.

Successful IO access was achieved in 146 neonates (91%). In 109 neonates (75%), success on the first attempt was achieved. Further details are presented in Figure 1. There was neither a significant difference in success rates between neonates with an age less than or greater than 24 h of life (*p* = 0.99) nor a significant difference in success rates according to gestational age (*p* = 0.67). Regarding the education level of the provider, there were no significant differences in IO overall success (*p* = 0.71), first-attempt success (*p* = 1.00), complication (*p* = 0.83), or severe complication (*p* = 0.39) rates between chief/senior physicians and resident/assistant physicians.

### Alternative access attempts

Data on alternative access attempts before IO decision-making were available for 137 neonates and are presented in Table 2. The number of attempts was (median) 2 for peripheral venous access (range 1–15) and 1 each for UVC and central venous access (both range 1–2). In 14 neonates, more than one type of alternative access was attempted. No significant differences in the number of peripheral vein access attempts were noted (*p* = 0.78) between experienced (chief/senior physician) and less experienced users (resident/assistant physician).

### Complications

Information about complications was available in 155 of the 161 patients. In 100 patients (65%) with successful IO access, no complications were observed. Of the 55 neonates with reported events, 46 (84%) were classified as minor, including 44 cases of misplacement in soft tissue and mild paravasation, one with healing deficiency and one with local swelling. Nine neonates with IO access (6%) experienced potentially severe complications, such as necrosis (*n* = 3; size of necrosis unknown), peripheral perfusion problems (*n* = 2), fracture (*n* = 1), broken IO needle (*n* = 1), osteomyelitis (*n* = 1), and soft tissue infection (*n* = 1). A trend was noted, but the presence of complications was not significantly related to GA (*p* = 0.06). Additionally, there was no significant impact of the education level of the provider on the overall occurrence of complications (*p* = 0.91) or the rate of severe complications (*p* = 0.39). Regarding the two age groups separately (infants < 24 h and infants aged between 1–28 days) the distribution of types of complications between the groups was similar with the exception that the two infection-related complications (osteomyelitis and soft tissue infection) both occurred in the group of infants aged 1–28 days.

### Preterm born infants

Regarding the subgroup of the 16 preterm infants, 10 were aged < 24 h, and 6 were aged between 1 and 28 days. IO was successfully established in 14 preterm infants. Three were successful on the first attempt with a GA of less than 30 weeks (25 + 6, 26 + 5, and 28 + 0 weeks). As with the overall group, in the subgroup of preterm infants the overall and first attempt success rates did not correlate with GA at birth (*p* = 0.93 and *p* = 0.27, respectively). Two of the four patients in which the EZIO needle was inserted manually were preterm (GA 26 + 5 and 28 + 0). Complications (2× misplacement in soft tissue, 1× extra/paravasation) among preterm infants occurred in 3 out of 15 infants (20%).

## Discussion

This prospective study on the use, feasibility and safety of IO access in neonates is the largest series published to date. We analysed data on 161 neonates with IO access up to 28 days after birth.

It is assumed that failure rates for IO access are greater in younger patients and, hence, are the greatest in the neonatal setting (17). Nevertheless, recent publications provide evidence for the successful use of IO access even in the youngest of patients (5, 7, 12) with success rates of 89 (7) and 75% (12) in neonates. In our study, successful IO insertion and infusion of medication was achieved in 91% of patients with success on the first attempt in 75%. The success rate in preterm infants was 88%, and IO access was even successfully established in three infants with a GA of less than 30 weeks. When considering GA, there was no significant impact on success rates. However, the group size of preterm infants may not have been large enough to show a significant effect and thus further studies specifically designed for preterm infants are warranted. Although data on birth weights were not available in our study, our results nevertheless indicate that IO access is feasible in neonates and might also be used below the recommended threshold of 3 kg. There is no information about previous training of the providers in the use of IO devices but the provider’s educational level did not impact on success rates. However, the high success rate found in this surveillance study must be interpreted with care due to the methodological limitations of potential selection and reporting bias. It is possible that more unsuccessful IO attempts were not reported, and the success rate presented here might be overestimated. Furthermore, it is unfortunately not known how many of the 345 paediatric departments generally participating in ESPED were actually actively involved in the survey. An additional bias therefore is possible. An indentation the predominant puncture site used in this analysis was the proximal tibia (96%), but other sites (distal tibia, distal femur, and proximal humerus) were also chosen. Eifinger et al. recently analyzed alternative sites for IO access in stillborn neonates and suggested the proximal humeral head as well as the distal femoral end as possible further puncture sites in neonates (18). Clearly, future studies are required to compare different puncture sites and success rates in the neonatal population.

In addition to continuous, effective ventilation of critically ill neonates, the time from the decision to the first infusion of medication or fluid is crucial in life-threatening situations. As previously published, in the resuscitation of children (mean age 2.1 years), the median time to IO access was 3 min (19). In simulated neonatal resuscitation, IO access was established in only 86 s (including equipment preparation) (20). These data are consistent with our real-life data with an estimated duration of less than 3 min in 79% of cases. Our data are based on retrospective estimations and therefore must be considered with caution. Future studies specifically designed to analyze implementation times for IO access in neonates are needed.

There was a high rate of previous attempts for alternative access routes before IO access in this study (in 80% of patients). As published elsewhere, the median time for successful placement of peripheral intravenous access in paediatric resuscitation was 8.7 min (19). In our study, the highest number of reported attempts for peripheral intravenous access was 15. The short implementation time for IO access indicates that the duration for vascular access implementation in neonates might be reduced rigorously by avoidance of several peripheral intravenous attempts and earlier use of IO instead.

A very low complication rate was reported in paediatric patients receiving IO access (3, 8). To date, safety data for IO access use in the neonatal setting is limited, but the risk of complications appears to be higher in younger patients (12, 17). Our study results confirm this assumption given that severe complications were observed in 6% of neonates. However, some of these complications (necrosis, fracture, broken IO needle, perfusion problems) might be acceptable in these life-threatening situations. Furthermore, the effect of proper and regular IO access training of medical teams on complication rates should be evaluated in future studies.

Although the ERC still recommends the use of an UVC as the first-line access in neonatal resuscitation immediately after birth, the alternative use of an IO access is mentioned for the first time in the recently published 2021 ERC newborn life support guidelines (1). Our current study underlines that IO placement during neonatal resuscitation is a widespread practice and demonstrates the feasibility of IO access in the population of neonates. This alternative is supported by a recent online questionnaire study, including 502 health care professionals, which revealed that UVC implementation during an emergency event was rated as very difficult to impossible in 60% by neonatologists and in 90% by non-neonatologists (21). Hence, taking our study results into account, we propose that in certain settings (beyond high-level neonatal units, prehospital or other difficult conditions, inexperienced teams, infant age above 24 h), IO access should be considered the first-choice access in the case of neonatal resuscitation.

Furthermore, the recent ERC guidelines recommend applying the paediatric life support (PLS) algorithm for all children aged 0–18 “except for ‘newborns at birth”’, for whom recommendations are found in the neonatal life support (NLS) (1). Therefore, for newborns requiring intensive care after the immediate postnatal period, according to PLS, IO access is recommended as the first choice for venous access. For critical events in the first 4 weeks of life, however, the probability is high that they fall under the responsibility of neonatal teams. Adversely, IO devices are still frequently unavailable in neonatal care units (20), and neonatal staff usually do not receive regular training in the use of IO devices. Consequently, regular role-appropriate training on IO access use in neonatal teams is urgently required and might further reduce complication rates.

Further studies of IO use in neonates analysing success and complication rates, needle type, method and location of insertion are needed to optimise practice in neonatal emergency situations.

## Conclusion

Within this surveillance study, IO access in neonates represents a feasible, safe and fast possibility for emergency vascular access. IO access should be available for time-sensitive emergencies at all neonatal sites. Neonatal departments must ensure that medical teams receive regular, interdisciplinary training in IO placement techniques.

## Data availability statement

The raw data supporting the conclusions of this article will be made available by the authors, without undue reservation.

## Ethics statement

The studies involving human participants were reviewed and approved by the Ethics Committee of the medical faculty of Ludwig-Maximilians-University, Munich Nr. 641-16 (19-12-2016). Written informed consent for participation was not required for this study in accordance with the national legislation and the institutional requirements.

## Author contributions

FH, SB, and MO conceived the study, designed the trial, and obtained research funding. FH, SB, MO, and DP supervised the conduct of the trial and data collection. FH, DP, ES, and J-CS managed the data, including data quality control. DG and DP provided statistical advice on the study design and analysed the data. ES, FH, DP, and MO drafted the manuscript. All authors confirm that they had full access to all the data in the study and accept responsibility to submit for publication and contributed substantially to its revision.

## Conflict of interest

Authors ES and J-CS are partners and CEOs of an Austrian training company that conducts paediatric and neonatal resuscitation training. The remaining authors declare that the research was conducted in the absence of any commercial or financial relationships that could be construed as a potential conflict of interest.

## Publisher’s note

All claims expressed in this article are solely those of the authors and do not necessarily represent those of their affiliated organizations, or those of the publisher, the editors and the reviewers. Any product that may be evaluated in this article, or claim that may be made by its manufacturer, is not guaranteed or endorsed by the publisher.
